# Effects of *Hedysarum* leguminous plants on soil bacterial communities in the Mu Us Desert, northwest China

**DOI:** 10.1002/ece3.6779

**Published:** 2020-09-21

**Authors:** Ziyuan Zhou, Minghan Yu, Guodong Ding, Guanglei Gao, Yingying He, Genzhu Wang

**Affiliations:** ^1^ Yanchi Research Station School of Soil and Water Conservation Beijing Forestry University Beijing China; ^2^ Key Laboratory of State Forestry Administration on Soil and Water Conservation Beijing Forestry University Beijing China

**Keywords:** desert, *Hedysarum* Linn., Leguminous plants, root endophytes, soil microbial communities

## Abstract

This study assessed the influence of rhizocompartment types (i.e., root, rhizosphere soil, root‐zone soil, and intershrub bulk soil) on the diversity of soil microbial communities under desert leguminous plant shrubs. Moreover, the influence and variations of soil physicochemical factors in interactions among leguminous plants, soil, and microbes were investigated. Both 16S rRNA high‐throughput genome sequencing and conventional soil physicochemical index determination were used to characterize both the bacterial diversity and soil physicochemical properties in the rhizocompartments of two *Hedysarum* species (*Hedysarum mongolicum* and *Hedysarum scoparium*) in the Mu Us Desert of China. All nutrient indices (except total phosphorus and available phosphorus) in rhizosphere soil were uniformly higher than those in both root‐zone soil and intershrub bulk soil (*p* < .05). The bacterial community diversity in the root, undershrub soil (i.e., rhizosphere and root zone), and intershrub bulk soil also showed significant differences (*p* < .05). The bacterial community in the root is mainly composed of Proteobacteria, Actinobacteria, Bacteroidetes, Tenericutes, and Chloroflexi, among which bacteria of the Proteobacteria genus are dominant. Root endophyte and rhizosphere soil microbiomes were mainly influenced by soil nutrients, while bacterial communities in root‐zone soil and intershrub bulk soil were mainly influenced by soil pH and NH_4_^+^‐N. The rhizocompartment types of desert leguminous plants impose a significant influence on the diversity of soil microbial communities. According to these findings, nitrogen‐fixing rhizobia can co‐exist with nonsymbiotic endophytes in the roots of desert leguminous plants. Moreover, plants have a hierarchical filtering and enriching effect on beneficial microbes in soil via rhizocompartments. Soil physicochemical factors have a significant influence on both the structure and composition of microbial communities in various rhizocompartments, which is derived from the interactions among leguminous plants, soil, and microbes.

## Graphical abstract

The rhizocompartment types of desert leguminous plants haves a significant influence on the soil physicochemical properties and the diversity of soil microbial communities. Desert leguminous plants have hierarchical filtering and enriching effect on the specific bacterial microbiome in soil via rhizocompartments. The niche differentiation among rhizocompartments under shrubs derived from the interactions among leguminous plants, soil, and microbes.

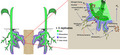

## 1 INTRODUCTION

In desert areas, the climate and environment are extremely harsh; the constant aridity, deficiency of soil nutrients and organic matter, and degradation of land all pose serious threats to human survival and biological diversity (Zhu, Li, Li, Liu, & Xue, 2010). In this context, the improvement of soil texture and fertility, and the restoration of ecological vegetation have become urgent issues (Wang et al., 2012). The genus *Hedysarum* Linn. comprises leguminous plants that are extensively distributed in deserts. Their roots are characterized by a strong sprouting ability, a deep taproot, high stress resistance, and high nitrogen‐fixing efficiency. The genus *Hedysarum* plays a vital role in improving soil fertility, fixing soil and sand, and restoring vegetation; these are pioneer plants, often used in the ecological restoration of deserts (Hou, He, Li, Wang, & Zhao, 2019; Xie, He, Wang, Hou, & Sun, 2017). *Hedysarum mongolicum* and *Hedysarum scoparium* are two species endemic to quicksand environments in Asian deserts with their main distribution in the desert areas of Northwest China (Sun et al., 2017; Xie et al., 2017). Plants of this species have well‐developed roots and can withstand both wind erosion and extreme aridity, thus making them effective pioneer species for vegetation restoration efforts in northwest China (Gao et al., 2014; Lai et al., 2016; Sun et al., 2017). In addition, *H. scoparium* can be used as forage grass to support animal husbandry, and as an oil extraction source of woody oil and fibrous plants. Yang et al. (2010) studied the effects of soil moisture on the growth of *Hedysarum* seedlings in desert areas and found that the rapid growth of roots during the seedling stage effectively adapts to extreme drought environments and controls the windward side of sand dunes, thereby slowing down the movement of sand dunes. Studies have also shown that *Rhizobium sullae* cannot survive independently under extreme drought or alkaline environments that *Hedysarum* plants can tolerate. When the bacteria establish a mutually beneficial symbiotic relationship with *Hedysarum* plants (Yates et al., 2015), they rely on the host plant to survive and in turn provide host plants with nitrogen for growth via nitrogen fixation (Sablok et al., 2017; Squartini et al., 2002).

The soil supports the growth of plants and supplies them with nutrients needed for growth. At the same time, with plants fixing external carbon sources, the physical, chemical, and biological characteristics of the soil are, in turn, improved (Pérez‐Bejarano et al., 2010). Some previous studies pointed out that the distribution and growth of vegetation communities in desert ecosystems are restricted, with varying degrees, by the soil microorganisms (Mengual, Schoebitz, Azcón, & Roldán, 2014; Saul‐Tcherkas & Steinberger, 2011). As a driving force in the transformation of soil organic matter and nutrients, soil microbes are pivotal in ensuring the stability and sustainability of ecosystems (Kennedy & Smith, 1995). Soil microbes are important components of belowground biota and are essential for a wide range of ecosystem processes such as decomposition, nutrient cycling, soil carbon storage, and maintenance of the soil structure (Kardol & Wardle, 2010; Zeller, Liu, Buchmann, & Richter, 2008; Zelles, 1999).

Rhizobia are Gram‐negative bacteria that are extensively distributed in soil. They can infect and live on leguminous plants, form nodules in their roots, supply the nitrogen nutrients needed by leguminous plants during growth through nitrogen fixation from soil and air, and build a mutualistic symbiotic relationship with leguminous plants (Batut & Boistard, 1994; He & Zhu, 1998). This interaction is based on unique transcription and the signal transmission of symbiotic genes of the host plant and rhizobia, which in turn trigger the corresponding organogenesis and adaptive physiological response of the host plant (Sablok et al., 2017). Various studies have shown that *R. sullae* is an efficient nitrogen‐fixing rhizobium that can enter the root system of the genus *Hedysarum* and establish a mutually beneficial symbiotic relationship with the host (Yates et al., 2015). Although *R. sullae* cannot survive in the extreme environment that the genus *Hedysarum* can tolerate, the bacteria can rely on the host plant to survive in an arid or alkaline environment (Sablok et al., 2017). And the interaction between such a wide range of plants and microorganisms mostly occurs in the rhizosphere soil, where plant root systems are in close contact with the soil.

In research on plant‐associated microbes, the diversity of rhizospheric microbes has increasingly become a focus of attention (Bulgarelli et al., 2012; Edwards et al., 2015; Lundberg et al., 2012; Philippot, Raaijmakers, Lemanceau, & van der Putten, 2013). Since they inhabit narrow rhizocompartments that surround plant roots, microbial communities strongly affect the growth and nutrient absorption of plants, and even the structure and distribution of plant communities (Hoeksema et al., 2010; Ke & Miki, 2015; Maron, Marler, Klironomos, & Cleveland, 2011). In turn, plants regulate the soil pH and the structure of rhizocompartments via root exudates, thus substantially influencing microbial communities in the rhizosphere and root‐zone soils (Gottel et al., 2011; Maron et al., 2011). In addition, numerous rhizospheric microbes can enter root tissues and form an endophyte microbiome, which may form a substantially different community composition from that of the rhizospheric microbiome (Lundberg et al., 2012; Xie et al., 2017). Hiltner has defined this differential phenomenon as the “rhizosphere effect” (Hartmann, Rothballer, & Schmid, 2008). Rhizocompartments constitute a special micro‐ecosystem. Differences in various soil factors among roots, rhizosphere soil, and nonrhizosphere soil can be used both as a reflection of the intensity of the rhizosphere effect and to characterize the degree of interaction between plants and soil. Assessment of these factors offers a feasible method for the quantitative study of bacterial diversity in the rhizocompartments of desert leguminous plants.

This study investigated the diversity of microbial communities of two *Hedysarum* Linn. desert leguminous plants and their undershrub rhizocompartments. The obtained data support desertification control, degraded land restoration and provide guidance for the development of future governance measures. The main hypotheses of this paper are as follows: (a) bacterial communities show hierarchical diversity and differential variations in undershrub and intershrub soils of desert leguminous plants; (b) the diversity of microbial communities in the rhizocompartments of desert leguminous plants is driven by interactions among soil factors, leguminous plants, and microbes in rhizocompartments.

## 2 METHODS AND MATERIAL

### 2.1 Research site

**FIGURE 1:**
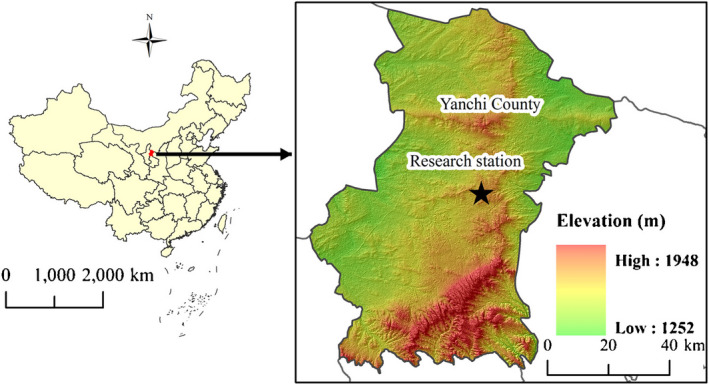
Location of the study area

This study was conducted at the Yanchi Research Station (37°04′–38°10′N, 106°30′–107°47′E) in Ningxia Province, northwest China, in June 2018 (Figure 1). The study site is located at the southern fringe of the Mu Us Desert, which is characterized by a typical semiarid continental monsoon climate with dry and warm climate throughout the year. The mean annual temperature is 8.1°C (range: −29.4°C to 37.5°C; relative humidity range: 49%–55%), and a mean annual rainfall of ~292 mm, 60%–70% of which falls between July and September (maximum in August). The local soil type is eolian sandy soil (Yu et al., 2015). The soil texture of the study area is sandy in the 0–1 m profile, with a mean bulk density of 1.5 g/cm^3^ (Lai et al., 2016). Ecological degradation in this area is mainly caused by overgrazing, climate change, and wind erosion, which has resulted in the degradation of arid grasslands into sandy land. The existing vegetation was established via aerial seeding (mainly *Artemisia ordosica*, *H. mongolicum*, and *H. scoparium*), seedling planting (*Caragana microphylla*), and natural restoration, which began in 2001 (Lai et al., 2016). Currently, the major dominant xeric shrub species in this area include *A. ordosica*, *H. mongolicum*, *Salix psammophila*, *H. scoparium*, and *C. microphylla*.

### 2.2 Sampling strategy and physicochemical analysis of soil

**FIGURE 2:**
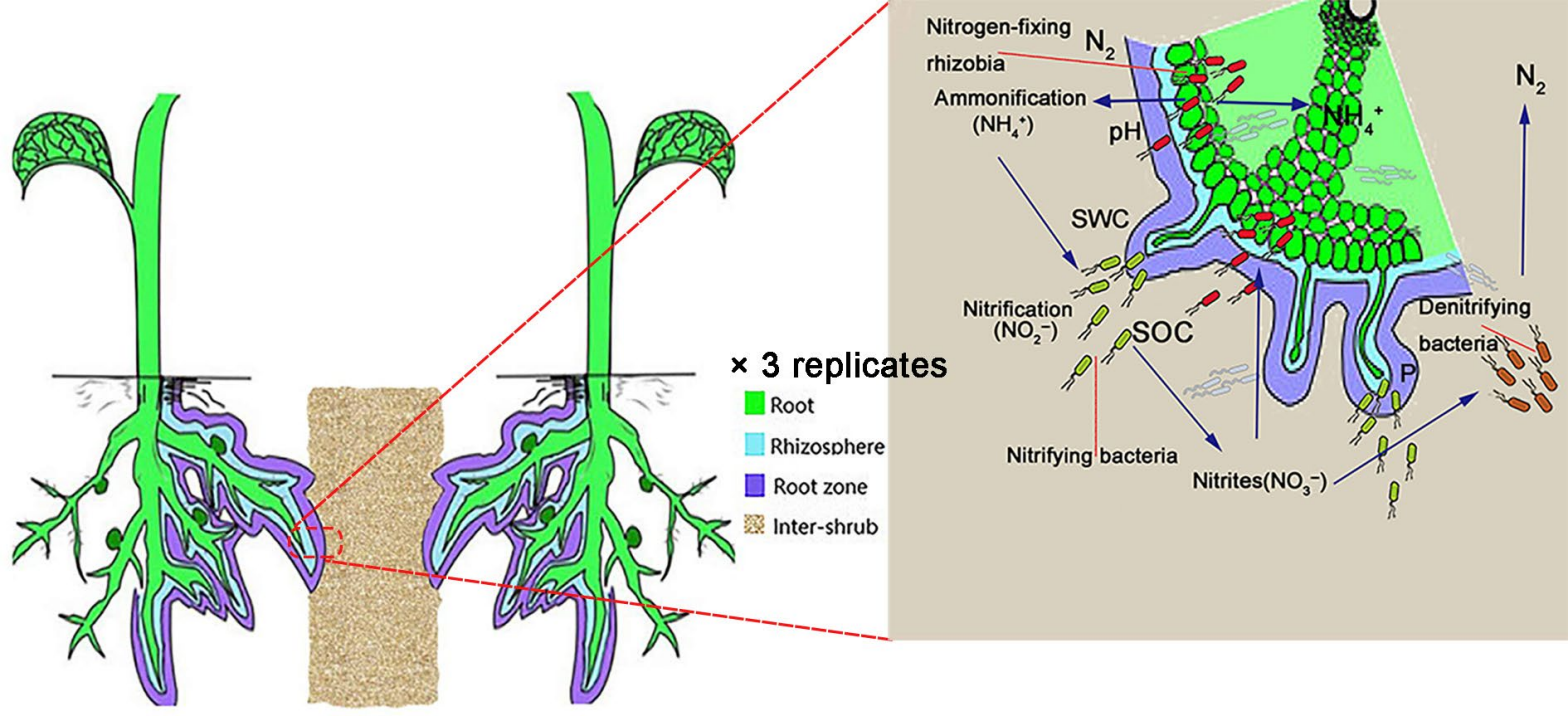
Flowchart of the experimental design and sketch of rhizocompartment types (i.e., roots, rhizosphere soil, root‐zone soil, and intershrub bulk soil)

The common dominant xeric leguminous shrub species of this area were selected for analysis, namely, *H. mongolicum* and *H. scoparium*. These two leguminous plants are both dominant xeric shrub species that naturally co‐occur and are widely distributed in the desert areas of Northwest China (including the study area). The two shrub species selected for this study were collected from a sample plot, formed via aerial seeding and natural enclosure; the adaptability of plants of these species to a desert environment and their ecological restoration effects have received close attention (Gao et al., 2014; Lai et al., 2016; Sun et al., 2017). The sample plants were mainly distributed on the sunny slopes of immobilized dunes that had formed via natural enclosure. Three sample plots (100 × 100 m, 100 m apart) were established for each species. For each shrub species, five shrubs were randomly selected from the immobilized sample plots and both plant root tissue and soil samples were collected. We had initially considered to include the legume nodule samples, but during the actual sampling, we found that the two *Hedysarum* spp. chosen, rarely have nodules in their root systems. Thus, we could not collect the nodules, as other studies have (Edwards et al., 2015; Xiao, Chen, et al., 2017). Occasionally, the nodule samples were available, but the 16S rRNA high‐throughput gene sequencing analysis of the collected samples showed that the composition of the nodule endophytic community and the bacterial community in the root system involved were not significantly different. We believe that this might be because (a) the extremely harsh desert environment makes it difficult for genus *Hedysarum* to form nodules; (b) the nodules we occasionally collected might not have been induced by rhizobia, but could have been enlarged hyperplastic nodules of plant root tissues, which form part of the root system; and (c) these samples were collected at the flowering stage of the plant; perhaps, there are fewer nodules established in the plants at this physiological stage. Based on the above reasons, we removed the comparison of nodule grouping data at the time of data analysis. Thus, we only analyzed the bacterial community diversity in the four rhizocompartments, including three types of undershrub sample (i.e., root, rhizosphere soil, and root‐zone soil) and intershrub bulk soil. (Figure 2). All samples were collected during the flowering stage of test plants in June 2018. Samples of the four rhizocompartment types were based on local conditions and were collected according to the sampling method described by Xiao, Chen, et al. (2017). After excavating to the roots, tweezers were used to pick up the soil particles adhering to the roots as rhizosphere soil to determine soil physicochemical properties. After that, roots were dug out and slightly shaken to remove large attached soil aggregates, and gloves were worn to collect large soil aggregates as root‐zone soil, which were then put into aseptic bags. Root samples were obtained from the secondary or tertiary branches of plant roots. Healthy and intact roots with an even thickness (5–8 cm) were removed and stored in sterile sample bags. Intershrub bulk soil was collected at the same sampling depth as root‐zone soil, 10–40 cm below intershrub bare soil. All collected samples were stored in preservation boxes with dry ice and brought back to the laboratory for subsequent treatment.

Root samples were first oscillated for 1 min in 50 ml centrifuge tubes containing 25 ml PBS buffer (PBS‐S; 130 mM NaCl, 7 mM Na_2_HPO_4_, 3 mM NaH_2_PO_4_, pH 7.0, 0.02% Silwet L‐77); then, two replicates were rinsed with new cleaning fluid at an interval of 5 min. The turbid liquid was then filtered through a 100‐µm nylon mesh cell strainer, and the remainder was centrifuged for 15 min at 3,200 × *g* to form a pellet, which contained fine sediment and microorganisms as the rhizosphere soil for molecular biological analysis. Sterile PBS buffer (25 ml) was added to a new sterile 50 ml tube, which contained the afore‐mentioned root samples, the mixture was vortexed, and this step was repeated until the PBS buffer was clear. The washed roots were placed into new centrifuge tubes containing 25 ml PBS buffer for ultrasonic oscillation for 5 min; an interval of 30 s was used between the two replicates. After discarding the fluid, clean root samples were collected. The above‐mentioned method for cleaning the root tissues has been verified by Xiao, Chen, et al. (2017), who proved that this method could effectively avoid the contamination and damage of plant tissue samples by subsequent cleaning procedures and could guarantee the ability of the thus treated samples to represent the rhizocompartment properties of the shrub species under investigation. Next, in reference to the sample treatment method described by Sun et al. (2017) for bacterial analysis, each collected plant species was subjected to three replicates according to its three sample plots. Samples from the rhizocompartments of the five shrubs in the same sample plot were mixed, and these mixed samples were established according to the four rhizocompartment types of each plant species. Each mixed soil sample was homogenized and filtered through a 2‐mm soil sieve to remove gravel and other impurities. Each mixed sample was divided into two subsamples: Part 1 contained 24 DNA samples (2 shrub species × 4 rhizocompartment types × 3 replicates), which were stored at −80°C for molecular biological analysis. Part 2 contained 18 soil samples (2 shrub species × 3 rhizocompartment types × 3 replicates), which were air‐dried for physicochemical analysis.

To determine the physicochemical properties of the soil samples, a conventional approach was adopted to quantify total soil organic carbon (SOC), total nitrogen (TN), total phosphorus (TP), available phosphorus (AP), ammonium nitrogen (NH_4_^+^‐N), nitrate nitrogen (NO_3_^−^‐N), and pH. SOC was quantified using the dichromate oxidation method (Walkley & Black, 1934). The TN concentration of soil samples was determined using a semi‐micro Kjeldahl Apparatus Nitrogen Autoanalyzer (Du, Pan, Li, Hu, & Wang, 2011). AP and TP were measured using an ultraviolet spectrophotometer (UV‐2550; Shimadzu). NH_4_^+^‐N and NO_3_^−^‐N were measured using the indophenol blue method and the hydrazine sulfate method, respectively, and soil pH was recorded on a 1:1 (10 g:10 ml) soil/distilled water slurry. The soil water content (SWC) was determined gravimetrically after oven drying at 105°C for 24 hr. All these methods were analyzed following international standard methods as adopted and published by the Institute of Soil Science, Chinese Academy of Sciences (1978).

### 2.3 16S rRNA gene amplification and sequencing

DNA was extracted from 0.5 g fresh samples using the EZNA Soil DNA Kit (Omega Bio‐Tek) following the manufacturer's instructions and was stored at −80°C for later use. When extracting tissues in the root, we only collected the internal tissues of the root system (excluding any tissues from the root epidermis) for subsequent analysis. After thawing on ice, extracted DNA samples were centrifuged separately and fully mixed. Sample quality was determined using NanoDrop, and 30 ng DNA was used for PCR amplification. PCR amplification was performed in 25‐μl reaction volumes containing 10 × PCR buffer, 0.5 μl dNTPs, 1 μl of each primer, 3 μl bovine serum albumin (2 ng/μl), 12.5 μl 2 × Taq Plus Master Mix, ultrapure H_2_O, and 30 ng template DNA. The PCR amplification program included initial denaturation at 94°C for 5 min, followed by 28 cycles of denaturing at 94°C for 30 s, annealing at 55°C for 30 s, and extension at 72°C for 60 s, followed by completion of the PCR amplification program at 4°C. The forward primer F799 (5′‐AACMGGATTAGATACCCKG‐3′) and the reverse primer R1193 (5′‐ACGTCATCCCCACCTTCC‐3′) were used to target the V5–V7 regions of 16S rRNA. It has been shown that the selection of hypervariable regions V5–V7 from root tissues for sequencing effectively decreased host contamination (Liu, Carvalhais, Schenk, & Dennis, 2017; Sánchez‐López et al., 2018; Zgadzaj et al., 2016). Both primers contained Illumina adapters, and the forward primer contained an 8‐bp barcode sequence unique to each sample. An agarose gel DNA purification kit (Axygen Biosciences) was used to purify and combine PCR amplicons. After purification, PCR amplicons were mixed at an equimolar concentration, followed by paired‐end sequencing, using the Illumina MiSeq sequencing system (Illumina) according to a standardized process.

After sequencing using the Illumina MiSeq system (Illumina), the results were stored in the Fastq format. The quantitative insights into microbial ecology (QIIME) software (version 1.8; http://qiime.org/) were used to analyze original Fastq files and conduct quality control according to the following criteria (Fierer, Hamady, & Knight, 2008; Miao et al., 2016): (a) Base sequences with a quality score < 20 at the read tails were removed, and the window was set to 50 bp; if the mean quality score in the window was < 20, the posterior‐end base sequences were discarded from the window; reads shorter than 50 bp were removed after quality control; (b) paired reads were assembled into one sequence according to the overlapping relationship between paired‐end reads (minimum overlapping length: 10 bp); (c) the maximum allowable mismatch ratio of the overlapping areas of assembled sequences was set to 0.1, and sequences that failed to meet this criterion in pairs were removed; (d) samples were distinguished according to barcodes and primers at the head and tail ends of sequences, and sequence directions were adjusted based on the number of mismatches allowed by barcodes; (e) different reference databases were selected to remove chimeras according to the type of sequencing data (using Usearch; version 8.1.1861; http://www.drive5.com/usearch/). Smaller‐length tags were discarded using Mothur to acquire clean tags of high‐quality sequences. The sequences were clustered into operational taxonomic units (OTUs) using UPARSE (version 7.1; http://drive5.com/uparse/) based on a 97% sequence identity cutoff (excluding single sequences). Thus, representative sequences and an OTU table were obtained (Edgar, 2013).

### 2.4 Statistics and analysis

The Vegan R package (v3.3.1; R Development Core Team, Vienna, Austria) was used to analyze the bacterial alpha diversity in the rhizocompartments of *Hedysarum* desert leguminous plants. To acquire the species taxonomy information corresponding to each OTU based on the QIIME platform (http://qiime.org/scripts/assign_taxonomy.html), the RDP classifier algorithm (http://sourceforge.net/projects/rdp‐classifier/) was used to comparatively analyze the representative sequences of OTUs and identify the species information of the different communities at various levels (kingdom, phylum, class, order, family, genus, and species). Differences in the OTU composition among rhizocompartments (based on Bray‐Curtis distance) were analyzed using one‐way analysis of similarity (ANOSIM) with 9,999 permutations. Principal component analysis (PCA) was used to evaluate the overall similarities in microbial community structure based on their Euclidean Distance. Hierarchical clustering of the samples based on Bray–Curtis dissimilarity was performed using QIIME. To generate statistics on the microbial taxa that contribute to the significant differences in microbial communities between root/rhizosphere soil and root‐zone soil, the OTUs of intershrub bulk soil were used as control, and Metastats (Mothur v.1.34.4; https://www.mothur.org) was employed to determine the significance (*p* ≤ .05) of intergroup differences in OTUs. To determine the co‐occurrence network pattern of bacterial communities in the rhizocompartments and the structural differences between bacterial communities in each niche, we conducted a spearman correlation analysis on the species of the 50 most abundant bacterial genus in each rhizocompartment. Subsequently, data with a correlation coefficient greater than 0.7 and *p*‐value < .05 were adopted, and the Gephi software was used for result visualization. Distance‐based redundancy analysis (db‐RDA) and the Mantel test were used to determine the major soil factors that shape microbial community structures. PCA, ANOSIM, RDA, and the Mantel test were implemented using the Vegan package in R 3.3.1 software.

The relative abundance data of all major contributing bacterial taxa filtered from the four rhizocompartments at phylum‐order‐genus levels were log‐transformed, thereby adhering to the requirements for normality of data and homogeneity of variance. The Shapiro–Wilk test and the Levene test were used to test for data normality and homogeneity of variance, respectively. Differences among treatments for diversity index, relative abundance data at phylum‐order‐genus levels, and soil physicochemical factors were analyzed using one‐way ANOVA, incorporating plant rhizocompartments as fixed factor. Post hoc comparison least significant difference (LSD) tests were performed at a 0.05 confidence level. All analyses were completed using SPSS 20.0 (SPSS Inc.).

## 3 RESULTS

### 3.1 Soil properties

In this experiment, soil samples were collected from undershrub (i.e., rhizosphere and root zone) and intershrub zones of *H. mongolicum* and *H. scoparium*. Variations were found in the soil physiochemical properties of rhizosphere, root‐zone soil, and intershrub bulk soil in both species. In both cases, the pH of the rhizosphere soil was lower than that of the root‐zone soil. The contents of all nutrients (except TP) in rhizospheres, root‐zone soil, and intershrub bulk soil showed significant differences (*p* < .05) (Table 1). A slight deficiency in NH_4_^+^‐N was observed in rhizosphere soils under both shrub species; however, for all other nutrient indices (except for TP and AP), values in rhizosphere soil were uniformly higher than those in both root‐zone soil and intershrub bulk soil (Table 1).

**TABLE 1:** Soil physicochemical properties of undershrub (rhizosphere, root zone) and intershrub bulk soil (*n = 3*)

Variable	HM			HS		
	Rhizosphere	Root zone	Bulk soil	Rhizosphere	Root zone	Bulk soil
SOC (g/kg)	1.99 ± 0.02^a^	1.75 ± 0.03^b^	1.58 ± 0.03^c^	1.79 ± 0.15^a^	1.67 ± 0.13^a^	1.51 ± 0.05^b^
pH	7.98 ± 0.13^b^	8.32 ± 0.18^a^	8.49 ± 0.11^a^	7.79 ± 0.14^b^	8.36 ± 0.21^a^	8.47 ± 0.25^a^
TN (k/kg)	0.32 ± 0.03^a^	0.21 ± 0.02^b^	0.12 ± 0.01^c^	0.30 ± 0.03^a^	0.24 ± 0.02^b^	0.21 ± 0.02^b^
NH_4_^+^‐N (mg/kg)	6.94 ± 0.02^c^	7.11 ± 0.02^b^	7.27 ± 0.05^a^	6.92 ± 0.05^c^	7.34 ± 0 0.12^b^	7.39 ± 0.03^a^
NO_3_^−^‐N (mg/kg)	16.52 ± 0.21^a^	13.14 ± 0.26^b^	11.77 ± 0.65^c^	15.37 ± 0.55^a^	12.18 ± 0.51^b^	11.89 ± 0.10^b^
TP (k/kg)	0.25 ± 0.02^a^	0.23 ± 0.02^a^	0.22 ± 0.02^a^	0.32 ± 0.02^a^	0.30 ± 0.01^a^	0.29 ± 0.01^a^
AP (mg/kg)	4.02 ± 0.20^a^	3.92 ± 0.13^a^	3.15 ± 0.20^b^	3.26 ± 0.11^a^	2.93 ± 0.18^b^	2.96 ± 0.07^b^
SWC (%)	8.08 ± 1.24^a^	6.01 ± 0.43^b^	4.82 ± 0.32^b^	6.07 ± 0.23^a^	4.67 ± 0.16^b^	3.98 ± 0.05^c^

Note

Data presentation: mean ± *SD*, *n* = 3; HM: *Hedysarum mongolicum*; HS: *Hedysarum scoparium*; bulk soil: intershrub bulk soil.

*Different letters* indicate significant differences between rhizosphere soil and root‐zone soil (*p* < .05).

Abbreviations: AP, available P; NH_4_^+^‐N, ammonium nitrogen; NO_3_^−^‐N, nitrate nitrogen; SOC, soil organic carbon; ST, soil temperature; SWC, soil water content; TP, total phosphorus.

Overall, the nutrient contents (except TP) of the undershrub soil were higher than those of intershrub soil (and significant differences were observed; *p < *.05).

### 3.2 Alpha and beta diversity of rhizocompartment bacterial communities

As shown in Table S1, all samples of the two *Hedysarum* Linn. leguminous plants achieved sound sequencing quality. The length distribution of high‐quality sequences (i.e., clean reads) was mainly concentrated at 360–380 bp, accounting for 88.05% of the total number of sequences (i.e., reads). Good's coverage index had a high comparability for the sequencing depth of the four rhizocompartment types (root, rhizosphere soil, root‐zone soil, and intershrub bulk soil), with a range of 95.57%–96.70%. This suggests that the sequencing depth was sufficient to reliably describe these plant rhizocompartment‐related bacterial communities (Table S1). The Good's coverage indices of root samples were also significantly higher than those of undershrub and intershrub bulk soil samples (*p* < .05) (Table S1). According to related data, the Chao1 and Shannon indices and the observed OTUs of microbial communities in undershrub soil samples are both significantly higher than those in plant root tissues (*p < *.05) (Table 2). By comparison, for the two desert leguminous plants, plant species and plant rhizocompartments jointly affect the alpha diversity of soil bacterial communities under and inter shrubs, but rhizocompartments play a leading role (Table 2).

**TABLE 2:** General features of the high‐throughput sequencing results (*n* = 3)

α‐diversity index		Chao1	Shannon	OTUs
Shrub species	Rhizocompartments			
HM	r	2,129.92 ± 136.28^b^	7.63 ± 1.04^b^	1,380.67 ± 189.01^b^
	rs	2,956.26 ± 248.03^a^	9.41 ± 0.22^a^	2,138.50 ± 204.54^a^
	rz	2,758.09 ± 57.84^a^	9.20 ± 0.22^a^	1,939.73 ± 84.27^a^
	b	2,375.57 ± 116.77^b^	8.74 ± 0.17^b^	1,707.47 ± 70.84^b^
HS	r	2,086.58 ± 200.28^b^	7.34 ± 0.30^b^	1,259.00 ± 149.94^b^
	rs	3,026.28 ± 131.57^a^	9.39 ± 0.29^a^	2,173.8 ± 191.76^a^
	rz	2,951.88 ± 61.28^a^	9.22 ± 0.10^a^	2,074.67 ± 78.36^a^
	b	3,056.71 ± 156.03^a^	9.28 ± 0.23^a^	2,169.53 ± 136.45^a^
*F*_shrub species_		1.95 ns	0.33 ns	0.70 ns
*F*_rhizocompartments_		14.88	26.55	23.2
*F*_shrub species×rhizocompartments_		15.87	11.56	16.84

Note

Data are presented as mean ± standard deviation, *n* = 3. HM: *Hedysarum mongolicum*; HS: *Hedysarum scoparium*.

r: root; rs: rhizosphere soil; rz: root‐zone soil; b: intershrub bulk soil.

*Different letters* signify significant differences among four rhizocompartments (*p* < .05). ns: no significant difference.

The last three rows represent the *F* values of the interactions between plant species/rhizocompartment types (shrub species × rhizocompartments).

**FIGURE 3:**
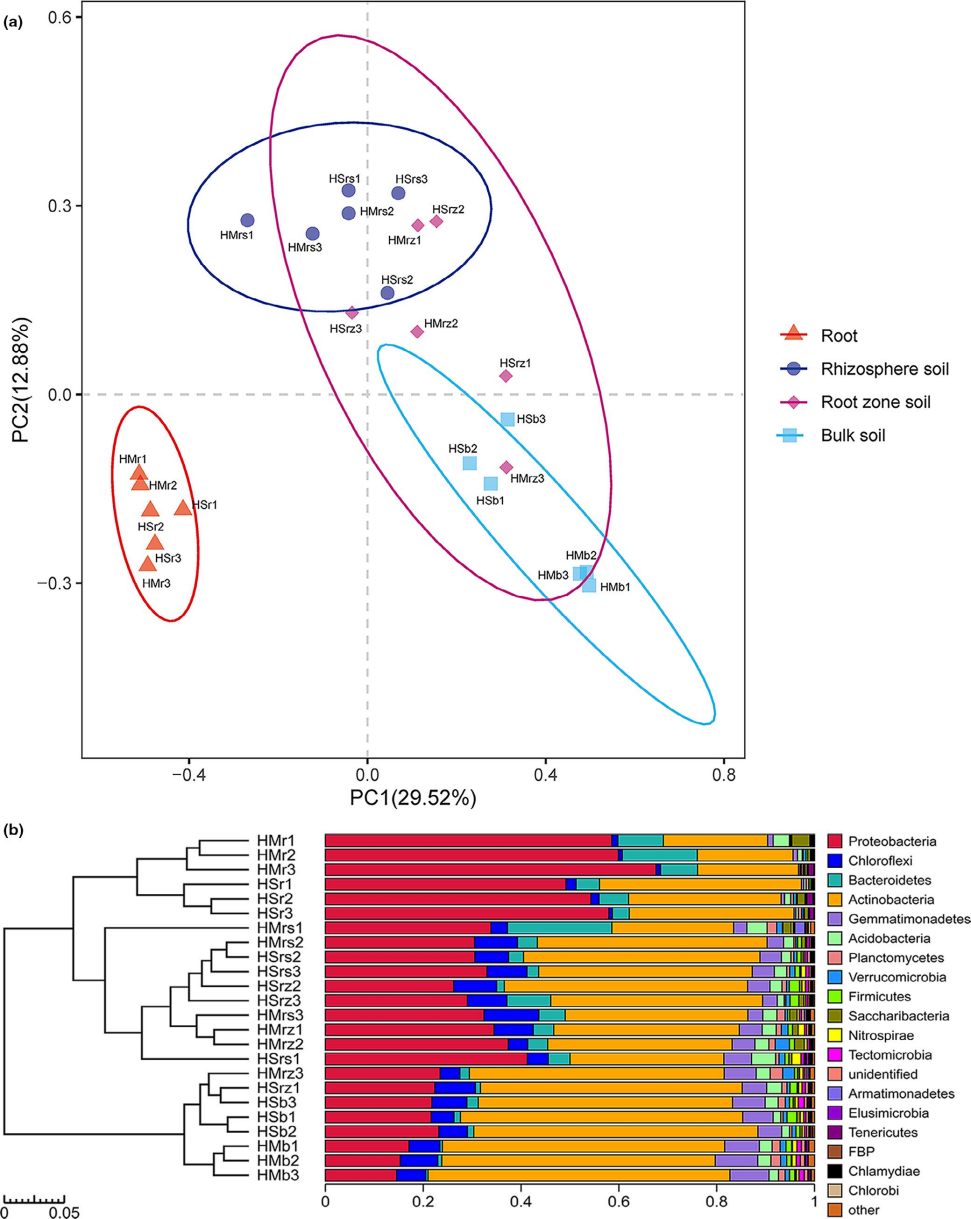
Compositions of bacterial communities driven by rhizocompartments at OTU and taxonomic levels. (a) Principal component analysis (PCA) on the compositions of bacterial microbes in the rhizocompartments of desert leguminous plants; (b) hierarchical clustering analysis on samples based on Bray–Curtis distance matrix. Abbreviations: HM: *Hedysarum mongolicum*; HS: *Hedysarum scoparium*; r: root; rs: rhizosphere soil; rz: root‐zone soil; b: intershrub bulk soil

PCA was used to rank the bacterial community compositions of the four rhizocompartments of the two *Hedysarum* species. The contributions of the primary and secondary principal coordinates were 29.52% and 12.88%, respectively. There were significant differences among the endophyte microbiomes of root and rhizosphere soil, root‐zone soil, and intershrub bulk soil (Figure 3). In summary, the samples from the same rhizocompartment sequencing group all clustered together, and the similarity level clustered above 95% (Figure 3a). Similar results were also obtained by grouping sources of various rhizocompartment samples and for hierarchical clustering based on a Bray–Curtis distance matrix at the phylum level, thereby verifying the clustering performance (Figure 3b). The results of hierarchical clustering analysis indicated that root samples of the two *Hedysarum* species clustered according to rhizocompartment type; the other three rhizocompartments (i.e., rhizosphere soil, root‐zone soil, and intershrub bulk soil) differed from root samples, and samples of undershrub soil did not cluster completely according to their respective rhizocompartment types (Figure 3b). Analysis of similarities (ANOSIM) showed that the bacterial community composition varied with both rhizocompartment type (*R* = .627, *p* = .001) and shrub species (*R* = .205, *p* = .009) (Figure S1).

### 3.3 Taxonomic composition of rhizocompartment bacterial communities and strategy for hierarchical filtration and enrichment of dominant microbial communities

**FIGURE 4:**
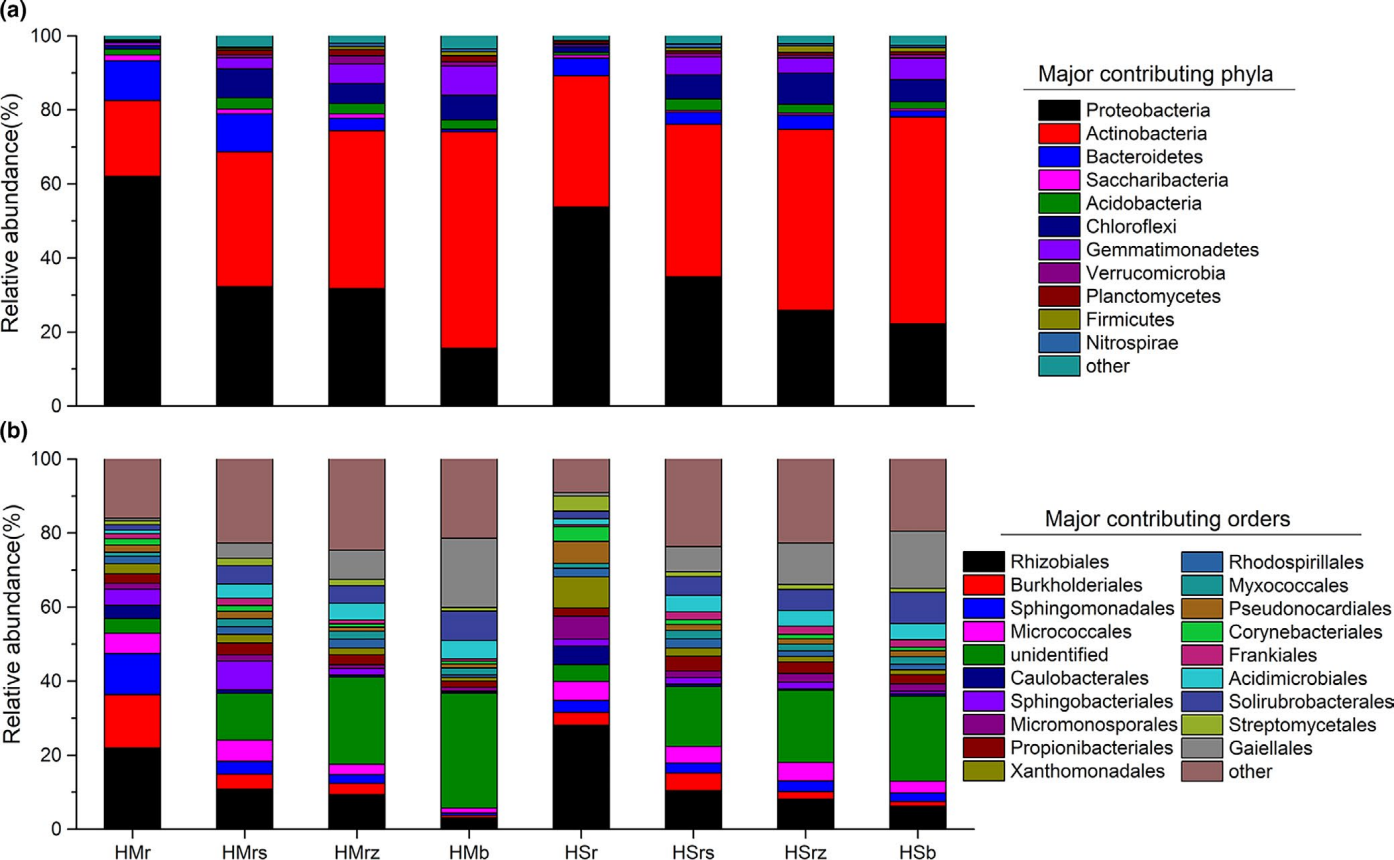
Microbial community compositions in the four rhizocompartments of two *Hedysarum* species at the phylum (a) and order (b) levels. Both groups only showed the bacterial phyla and orders with relative abundances > 0.1% and > 3%, respectively; those with lower relative abundances are included in “others.” For abbreviations, please refer to Figure 3

In the four rhizocompartments of the two *Hedysarum* species, bacterial phyla mainly consisted of Proteobacteria, Actinobacteria, and Bacteroidetes (relative abundances > 1%), which accounted for > 90% of the total bacterial composition (Figure 4 and Table S2). The four investigated rhizocompartments had a significant influence on the relative abundances of major bacterial phyla. Proteobacteria showed a significant trend of hierarchical enrichment in the order of intershrub bulk soil < undershrub soil < root (*p < *.05), while Actinobacteria showed the opposite trend (Figure 4).

For *H. mongolicum*, the major dominant bacterial orders of root endophyte microbes consisted of Rhizobiales, Burkholderiales, and Propionibacteriales. The relative abundances of Rhizobiales, Burkholderiales, Propionibacteriales, and Caulobacterales in microbes also showed a significant overall trend of hierarchical enrichment and filtration following the order of intershrub bulk soil < undershrub < root (*p < *.05) (Figure 4 and Table S2). At the genus level, Rhizobiales had a relative abundance of 22% in roots, mainly consisting of *Rhizobium* and *Bosea* (Table S2). For *H. scoparium*, the major dominant bacterial orders of root endophyte microbes consisted of Rhizobiales, Burkholderiales, and Propionibacteriales. At the genus level, Rhizobiales genera had a relative abundance of 28% in roots, mainly consisting of *Rhizobium* and *Bradyrhizobium* (Table S2).

**FIGURE 5:**
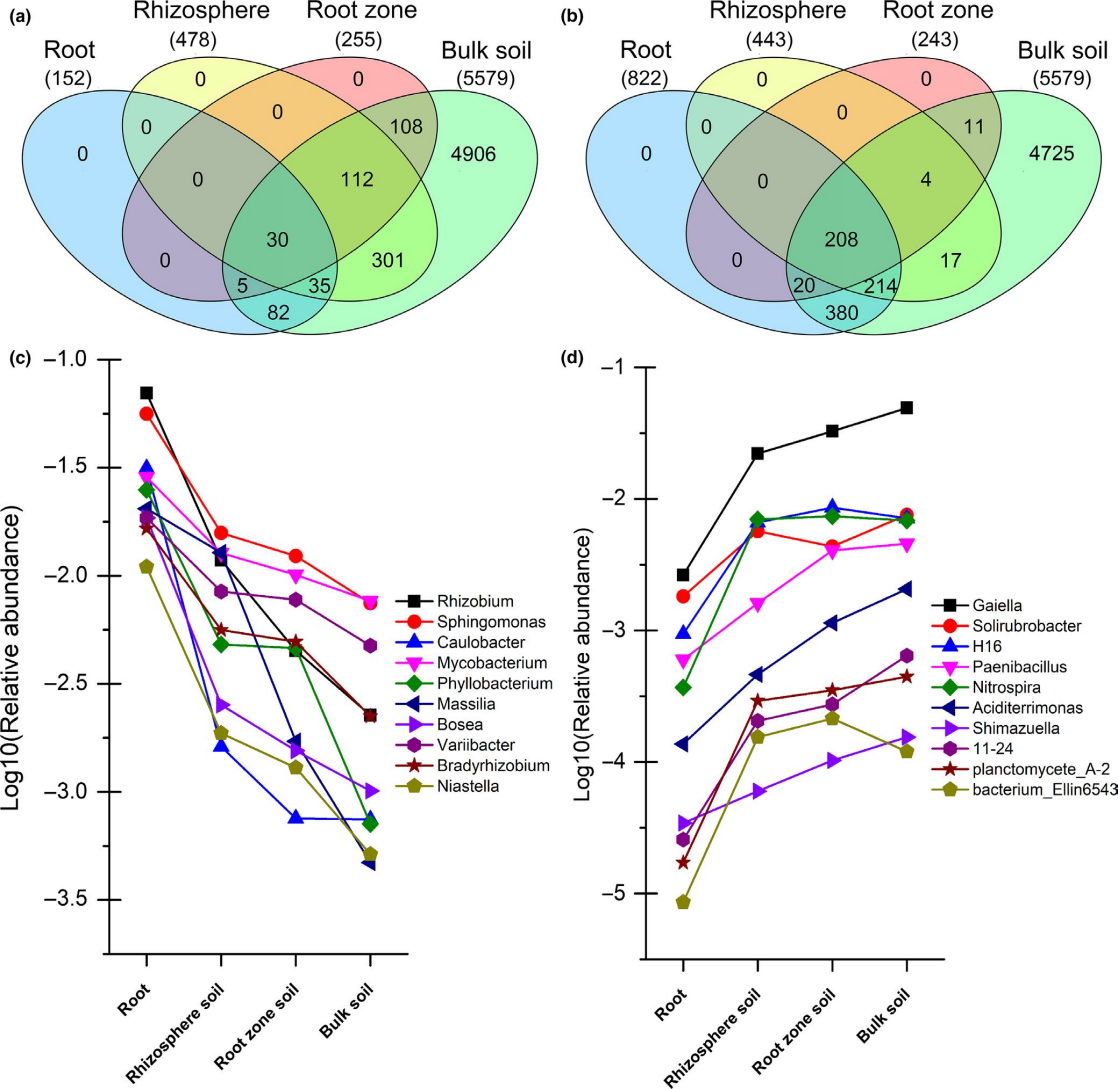
Venn diagrams of the significantly enriched (a) and reduced (b) operational taxonomic units (OTUs) in the other three rhizocompartments compared with intershrub bulk soil under *Hedysarum* desert leguminous shrubs. (c) Bacterial genera corresponding to relatively enriched OTUs; (d) bacterial genera corresponding to relatively reduced OTUs

The bacterial communities in the rhizocompartments all originated from soil; however, there are varying degrees of significant differences between the bacterial communities in the four rhizocompartments. This identified OTUs with significant enrichment or reduction in intershrub bulk soil relative to the other three rhizocompartments. As shown in Figure 5, the circles represent various rhizocompartment types, and the overlapping values represent the numbers of common enriched or reduced OTUs among various rhizocompartment types. Under the two *Hedysarum* species, compared with intershrub bulk soil, the other three rhizocompartments (i.e., root, rhizosphere soil, and root‐zone soil) had 152, 478, and 255 OTUs showing relative enrichment, and 822, 443, and 243 OTUs showing relative reductions, respectively (Figure 5). Based on Venn diagrams of the significantly enriched and reduced OTUs, the relatively enriched and relatively reduced OTUs showed maximum overlapping in rhizosphere soil and root samples, respectively.

Using intershrub bulk soil as control, the enriched OTUs in other rhizocompartments showed different degrees of overlapping. The 152 OTUs that were relatively enriched in the roots showed a consistent enrichment effect with 65 OTUs from rhizosphere soil and 35 OTUs from the root‐zone soil. The 478 relatively enriched OTUs in rhizosphere soil showed a consistent enrichment effect with 142 OTUs from the root‐zone soil. The other three rhizocompartments jointly showed an enrichment effect over 30 OTUs relative to intershrub bulk soil (Figure 5a). Statistical analysis on the 10 relatively abundant dominant bacterial genera corresponding to OTUs, recording a relative enrichment effect among the four rhizocompartments, indicated that these dominant bacterial genera manifested a trend of enrichment toward roots (*p* ≤ .05) (Figure 5c).

In contrast, the 822 OTUs that showed relative reduction in the roots manifested a consistent reduction effect with 422 OTUs from the rhizosphere soil and 228 OTUs from root‐zone soil. The 443 relatively reduced OTUs in the rhizosphere soil showed a consistent reduction effect with 212 OTUs from the root‐zone soil. There were 208 OTUs over which the other three rhizocompartments jointly showed a reduction effect relative to intershrub bulk soil (Figure 5b). Similarly, statistical analysis on the 10 relatively abundant dominant bacterial genera corresponding to the OTUs revealed a relative reduction effect among the four rhizocompartments, further indicating that these dominant bacterial genera all manifested a trend of reduction toward roots (*p* ≤ .05) (Figure 5d).

### 3.4 Rhizocompartments bacterial community co‐occurrence network analysis

**FIGURE 6:**
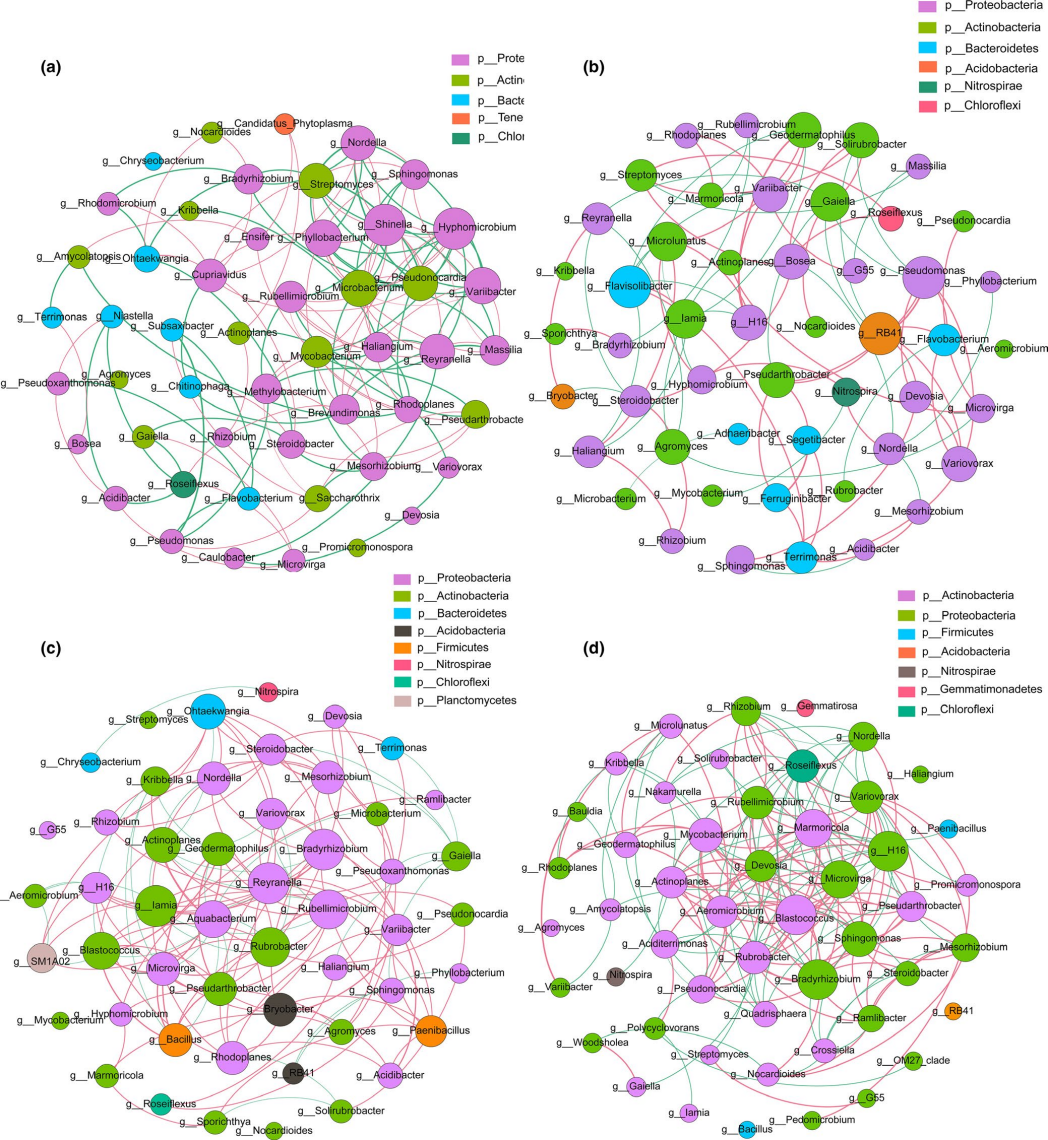
Species co‐occurrence network analysis of bacterial communities in four rhizocompartments. The red line indicates a positive correlation, while the green line indicates a negative relationship. The larger the circle of a species, the more the connections of the circle, and the more significant its correlation with other species, which represents the core species in the correlation. (a) co‐occurrence network of bacterial communities in the root, (b) co‐occurrence network of bacterial communities in the rhizosphere soil, (c) co‐occurrence network of bacterial communities in the root zone, (d) co‐occurrence network of bacterial communities in the intershrub bulk soil

**FIGURE 7:**
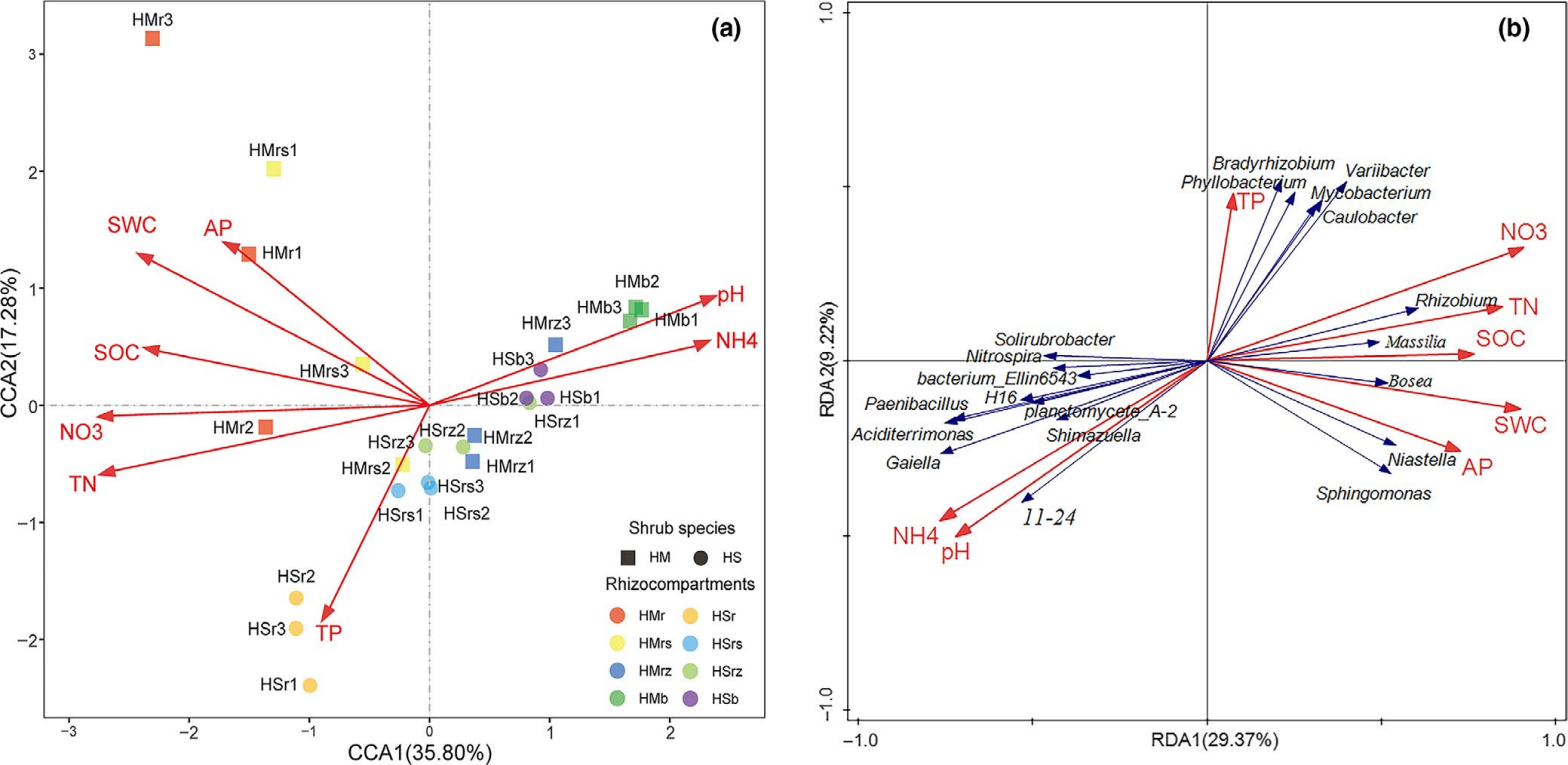
Redundancy analysis (RDA) on the bacterial communities in rhizocompartments and soil factors. (a) Effects of soil factors on the samples of four rhizocompartment types under two leguminous shrub species. (b) Effects of soil factors on the 20 relatively enriched or reduced bacterial genera in four rhizocompartments under two leguminous shrub species. For abbreviations, please refer to Figure 3. The significance test data of the CCA/RDA model, axes, and terms are listed in Table S3

From our co‐occurrence network analysis results, we could see that the bacterial community in the root is mainly composed of Proteobacteria, Actinobacteria, Bacteroidetes, Tenericutes, and Chloroflexi, among which bacteria of the Proteobacteria genus are dominant. In the co‐occurrence network structure of the bacterial community within the root at the genus level, *Hyphomicrobium, Shinella*, and *Phyllobacterium* showed a broad and robust correlation with other genera (Figure 6). Interestingly, network nodes linked to *Phyllobacterium* as the core are negatively related to bacteria genera affected by NH^4+^‐N and pH and are positively associated to bacterial genera affected by other nutrients (Figures 6, 7). Compared with bacterial communities in the root, core nodes from Actinobacteria were significantly higher in undershrub soil and intershrub bulk soil. *Flavisolibacter* and *Flavobacterium*, two Bacteroidetes genera, exhibited a wide range of strong correlations, especially in rhizosphere soil. Bacterial communities in the root zone had the most complex composition at the phylum level, and bacteria genus belonging to Acidobacteria and Planctomycetes also showed strong correlations. Bacterial communities in the intershrub bulk soil were mainly composed of genera from the Actinobacteria phylum, which occupy the majority of core nodes in the network (Figure 6).

### 3.5 Relationships between rhizocompartment bacterial communities and soil factors

In the investigated desert environment, the soil factors that affect the root and rhizosphere soil bacterial communities of the two *Hedysarum* plants are TN, NO_3_^−^‐N, AP, TP, SOC, and SWC, while factors that affect the bacterial community in the root zone and the intershrub bulk soil are NH_4_^+^‐N and pH (Figure 7 and Table S3). Among these soil factors, TN, NO_3_^−^‐N, AP, and SOC are positively correlated with SWC, while TP, NH_4_^+^‐N, and pH are negatively correlated with SWC (Figure 7). Among the four rhizocompartment bacterial communities, the root endophyte and rhizosphere soil microbiomes were mainly influenced by soil nutrients, especially nitrogen, while the communities in the root‐zone soil and intershrub bulk soil were mainly influenced by soil pH and NH_4_^+^‐N (Figure 7a). The 10 relatively enriched bacterial genera (Figure 5c), which underwent hierarchical filtration and enrichment through intershrub bulk soil to roots of legume plants, were mainly influenced by SWC and soil nutrients. The 10 relatively reduced bacterial genera (Figure 5d), which were relatively depleted in roots compared with the other three rhizocompartments, were mainly influenced by soil pH and NH_4_^+^‐N (Figure 7b). Mantel test results indicated that all nutrient indices (except TP and AP), pH, and SWC were significantly correlated with the microbial communities (*p* < .05) (Table 3).

**TABLE 3:** Correlation between bacterial community and soil properties as shown by the Mantel test

Soil properties	pH	TN	N‐NH_4_^+^	N‐NO_3_^−^	SOC	TP	AP	SWC
*r*	0.2849	0.353	0.2318	0.4256	0.2988	0.0318	0.1261	0.3334
*P*	0.002	0.003	0.016	0.001	0.001	0.314	0.066	0.006

Note

Bold type indicates significant difference (*p* < .05).

## 4 DISCUSSION

### 4.1 Composition of root endophytes

This study demonstrated that nitrogen‐fixing bacteria occupied a dominant position in the roots of xeric leguminous plants, but also that many non‐nitrogen‐fixing microbes were present. These results confirmed that nitrogen‐fixing rhizobia co‐exist with other bacteria in the roots of desert xeric leguminous plants, which is consistent with previously reported results, where isolated cultures were used to assess the *Rhizobium* strains of leguminous plants (Sablok et al., 2017; Toniutti et al., 2017; Yates et al., 2015). This study showed that in addition to dominant nitrogen‐fixing rhizobia, there were also other, nonsymbiotic endophytes in the roots of *Hedysarum* plants. It has been reported that *Burkholderiales* (Deng et al., 2011; Moulin, Munive, Dreyfus, & Boivin‐Masson, 2001), *Massilia* (Ofek, Hadar, & Minz, 2012), and *Phyllobacterium* (Liang et al., 2019) detected in the roots of leguminous plants can potentially promote plant growth. In addition, *Phyllobacterium* has also been detected in the roots of *Lupinus micranthus* in Tunisia, indicating that this bacterial genus can boost the rapid nodulation of this Tunisian xeric leguminous plant (Msaddak et al., 2018). It has also been reported that endophytic bacteria, which are known to exist in plant tissues, can facilitate plant growth (Rajendran, Sing, Desai, & Archana, 2008; Rivas et al., 2002) and that other root endophytes co‐inoculated with rhizobia can accelerate the growth and nodulation of leguminous plants (Chen et al., 2015; Xu, Zhang, Wang, Chen, & Wei, 2014). Together, these results demonstrate that nonsymbiotic endophytes in roots may coordinate with nitrogen‐fixing rhizobia and exert various functions.

### 4.2 Hierarchical filtration and enrichment strategy of rhizocompartments

The numbers of significantly enriched OTUs followed a decreasing hierarchical trend in the order of root < undershrub soil < intershrub bulk soil (*p* < .05). The majority of OTUs that were relatively decreased in roots also showed relative reductions in rhizosphere soil and root‐zone soil (Figure 5). This phenomenon can be considered as a hierarchical filtration strategy of desert leguminous plants on soil microbes (Xiao, Chen, et al., 2017). In the four rhizocompartments, bacteria were assessed at the phylum, order, and genus levels. The relative abundances of Proteobacteria, Rhizobiales, and other specific dominant bacterial taxa indicated an overall hierarchical enrichment trend in the order of intershrub bulk soil < undershrub soil < root (*p* < .05) (Figure 4). With respect to the mutualistic symbiotic relationship of rhizobia with leguminous plants, the enrichment trends discussed above suggest that the roots of desert leguminous plants exert a hierarchical enrichment and filtration effect for specific beneficial bacteria in the soil (Table 2, Figure 5). A similar hierarchical trend was also observed in the rhizosphere soil, root surface, and roots of rice (Edwards et al., 2015), as well as in the rhizosphere soil and internal root tissues of *Arabidopsis thaliana* (Lundberg et al., 2012). According to studies on the diversity of the rhizospheric microbiota of field‐grown corn, significant differences exist between undershrub soil and intershrub soil in terms of both the enrichment and diversity of bacteria and the relative abundances of bacterial groups (Peiffer et al., 2013).

Interestingly, the structural composition of microbial communities from rhizocompartments mainly consisted of Proteobacteria and Actinobacteria, both of which presented opposite enrichment trends and filtration strategies among various rhizocompartments (Figure 4). The relative abundances of Proteobacteria in the bacterial communities of rhizosphere soil are significantly related to the carbon‐nitrogen ratio of soil and the variability of the ammonium nitrogen pool; moreover, the significance level is higher than that of any other environmental variable in soil (Nemergut, Cleveland, Wieder, Washenberger, & Townsend, 2010). Specifically, the undershrub enrichment trend of Proteobacteria toward roots is positively correlated with the available carbon pool of soil; furthermore, Proteobacteria are closely related to heterotrophic N‐fixers, meaning that their presence can promote the utilization of ammonium nitrogen (NH_4_^+^ pools) (Nemergut et al., 2010). In contrast, the relative abundance of Actinobacteria is positively correlated with the pH of soil (Lauber, Hamady, Knight, & Fierer, 2019; Nemergut et al., 2010). This may explain why a slight deficiency in NH_4_^+^‐N was observed in rhizosphere soils under both shrub species (Table 1). Another study argued that the undershrub enrichment of Bacteroidetes is a result of their quick utilization of soil organic matter (Acosta‐Martinez, Dowd, Sun, Wester, & Allen, 2010). A number of studies have also obtained similar results about the variations of the structural composition of Proteobacteria and Actinobacteria in different ecological niches. This theory suggests that these reported variations may be the result of the differentiation of specific bacterial taxa in different ecological niches as well as the active filtration of bacterial groups by host plants; an alternative explanation is the opportunistic colonization of suitable ecological niches by specific bacteria (Beckers, Op De Beeck, Weyens, Boerjan, & Vangronsveld, 2017; Bulgarelli et al., 2013; Hacquard et al., 2015).

Overall, the nutrient content of the rhizosphere soil was higher than that of intershrub soil (*p < *.05). Enrichment trend analysis of soil nutrients under the influence of shrub roots obtained similar results (Philippot et al., 2013). Both wild (*Glycine soja*) and cultivated (*G. max*) soybeans had higher alpha diversity of bacterial communities in the rhizosphere compared with intershrub bulk soil, suggesting that the nutrients enriched in the undershrub soil are needed by plants during growth (Chang et al., 2018). In the Mu Us Desert in Ningxia Province, China, the nutrients in the undershrub and intershrub soils of three different xeric shrub species (i.e., *A. ordosica*, *S. psammophila*, and *C. microphylla*) have also revealed a similar trend of enrichment from intershrub soil to undershrub soil (Sun et al., 2017).

### 4.3 Interactions among desert leguminous plants, soil, and microbes

In this study, while soil pH mainly influenced the bacteria in the root‐zone soil and intershrub bulk soil, its influence on the bacteria in root endophytes was relatively insignificant (Table 3 and Figure 7). According to previous research, the influence of soil pH on the microbes of rhizosphere soil of soybean and alfalfa exceeded that of soil nutrients (Xiao, Fan, Wang, Chen, & Wei, 2017). Related studies also revealed an imbalance in the absorption of cations and anions by plant roots as the primary reason for variations in rhizosphere soil pH. The reasons for the decrease of plant rhizosphere soil pH include (Hinsinger, 2001): (a) imbalanced absorption of cations and anions; (b) secretion of organic acids by plant roots; (c) generation of CO_2_ by root cell respiration; (d) organic acids and CO_2_ generated by rhizospheric microbial activities; and (e) active secretion of H^+^ by roots. The pH and the NH_4_^+^‐N content was lower in rhizosphere soil of both plant species than in root‐zone soil and intershrub bulk soil, which is possibly because of the symbiotic nitrogen fixation between *Hedysarum* species and rhizobia and the use of NH_4_^+^‐N as their main nitrogen source. As a result, the absorption of cations played a dominant role, and the roots excreted more H^+^ than HCO_3_^−^ and OH^−^ to maintain a charge balance within the plants; this caused a decrease of rhizosphere soil pH relative to the pH of root‐zone soil. The local soil is mainly alkaline, and the absorption of NH_4_^+^‐N by leguminous plants and rhizobia for nitrogen fixation is very limited and exerts a small influence on undershrub soil. While the undershrub soil of leguminous *Hedysarum* species is largely alkaline, the rhizosphere soil pH is lower than the root‐zone soil pH (Table 1).

In contrast, only few soil microbes absorb and assimilate NO_3_^−^‐N; in the co‐existence of NO_3_^−^‐N and NH_4_^+^‐N, the latter inhibits the absorption of NO_3_^−^‐N by microbes, since the assimilation of NO_3_^−^‐N consumes energy (Wickramasinghe, Rodgers, & Jenkinson, 1985). From the deepening of rhizocompartments from intershrub bulk soil to roots, the absorption and utilization of nutrients by roots, as well as the activity of soil microbes, are gradually strengthened. This results in a decrease of the NH_4_^+^‐N content and has a significant influence of NO_3_^−^‐N content in rhizosphere soil (Table 1). In addition, the relative increase of soil pH can increase the NH_4_^+^‐N yield in soil, mainly because a higher pH improves the solubility of organic matter; this provides many matrices rich in carbon and nitrogen for the growth of microbes and thus promotes the mineralization of nitrogen (especially in the form of NH_4_^+^‐N) (Narteh & Sahrawat, 1997). According to previous studies, such changes in pH and nitrogen are related to the activities of soil microbes; during nitrogen assimilation, soil microbes usually show a preference for NH_4_^+^‐N (Jenkinson & Parry, 1989) and can more efficiently absorb and utilize NH_4_^+^‐N than NO_3_^−^‐N (Recous, Mary, & Faurie, 1990). Because of this preferential adsorption by nitrogen‐fixing microbes, NH_4_^+^‐N is immobilized in large quantities, and the NH_4_^+^‐N content of rhizosphere soil is significantly lower than that of root‐zone soil and intershrub bulk soil.

Some studies have pointed out that organic carbon is the main driving factor for changing the soil microbial community structure (Shen, Zhang, Guo, Ray, & He, 2010), and this effect is mainly caused by labile organic carbon in the soil (Eilers, Lauber, Knight, & Fierer, 2010). The organic carbon in the soil of the desert area is mainly composed of labile organic carbon decomposed from plant leaves and root litters (Liu et al., 2015). Similarly, our data showed that the content of SOC in the rhizosphere soil of the two species of genus *Hedysarum* was significantly higher than that of the intershrub bulk soil (Table 1). The Mantel test results also showed that SOC significantly affected the structure of the bacterial community in the four root rhizocompartments under desert shrubs (*r* = .2988, *p* = .001; Table 3). Fierer et al. (2008) reported that high SOC content under xerophytic shrubs might result in significant enrichment of Proteobacteria (mostly Alphaproteobacteria and Gammaproteobacteria). The changes in their relative abundance might be related to the increase in the content of labile carbon substrates in the soil. Some specific bacterial groups from the classes Alphaproteobacteria and Gammaproteobacteria play significant roles in promoting the recycling and utilization of SOC and TN in ecosystems (Kersters et al., 2006). Besides, studies have shown that soil microorganisms can transform soil‐bound P in soil into water‐soluble P and thus promote the absorption of P by plants (He & Zhu, 1998). In legume plants, P is also associated with complex signal transduction, energy conversion, respiration, and nitrogen fixation (Khan, Zaidi, Ahemad, Oves, & Wani, 2010). Studies have found that the relative abundance of phosphate‐solubilizing bacteria in the rhizosphere is far greater than that in intershrub soil, which can be explained by the faster consumption of P in the rhizosphere (Laheurte & Berthelin, 1988; Pande, Pandey, Mehra, Singh, & Kaushik, 2017). On the other hand, the effectiveness of soil moisture is a key factor that limits the diversity and productivity of plant communities in desert areas, because it can affect the quality and quantity of the plant litters and exudates entering the soil decomposed by soil microorganism. Also affected is the conversion efficiency of water‐soluble soil nutrients, which in turn affects the overall changes in soil texture (Bai et al., 2008; Sun et al., 2017). Therefore, under *H. mongolicum* shrubs (i.e., rhizosphere and root zone), a high diversity of bacterial communities was observed (*p* < .05; Table 2), and the bacterial communities of the four rhizocompartments were significantly affected by each nutrient element (except for P) (*p* < .05; Table 3 and Figure 7).

The pH of soil has an important influence on its chemical, physical, biological, and other processes, especially on nitrogen cycling (Fan et al., 2018). Baggs, Smales, and Betaman (2010) have probed into the influence of long‐term and short‐term pH differences on N_2_O emission from soil, as well as on N_2_O emission from microbial sources (ammoxidation and denitrification). According to their findings, the ammoxidation and nitrate nitrogen dissimilation (mainly denitrification) processes in soil generate N_2_O (Baggs, 2008), and variations of soil pH dominate N_2_O emission from microbial sources (ammoxidation and denitrification). Furthermore, in a comparison of long‐term soil pH differences, pH plays the most significant role, which can reflect the adaptability of microbial communities to their current habitat (Baggs et al., 2010). According to Hartman, Richardson, Vilgalys, and Bruland (2008), pH is the optimal factor to predict variations of bacterial communities in soil, and the authors have also observed variations of the relative abundances of Acidobacteria and Actinobacteria across the pH scale at the phylum level (Hartman et al., 2008). Other studies have also pointed out that variations of the relative abundances of Acidobacteria, Actinobacteria, and Bacteroidetes in soil microbial communities are mainly driven by soil pH, which further affect the overall structural variability of bacterial communities (Lauber et al., 2019). According to the findings of a recent study that investigated the bacterial communities in poplar roots, pH has a significant influence on the genotypes of both bacterial and fungal communities, as well as their colonization and distribution in plant roots and stems (Wang et al., 2019). As a result, microbes in root‐zone soil and intershrub bulk soil are mainly influenced by pH and NH_4_^+^‐N.

In summary, these results demonstrate that interactions occur among plants, soil, and specific microbes based on the enrichment and absorption of nutrients by rhizocompartments. These interactions affect the diversity of soil microbial communities and change the physicochemical properties of undershrub soil and intershrub soil. However, soil physicochemical factors also have a significant influence on the structure and composition of microbial communities in various rhizocompartments.

## 5 CONCLUSIONS

This study revealed that the soil physicochemical factors (especially the pH) have a significant influence on the structure and composition of microbial communities in various rhizocompartments, and this influence is derived from interactions among leguminous plants, soil, and microbes. The variations of bacterial diversity in the undershrub rhizocompartments of the two leguminous plants *Hedysarum* Linn. and *H. mongolicum* were consistent with that of *H. scoparium* with regard to the variations of bacterial diversity in undershrub rhizocompartments. This study explored the effect of leguminous plants on the microbial communities in desert landscapes and offered a microbe‐associated reference for evaluations of the restoration of desert vegetation.

## CONFLICT OF INTEREST

The authors declare no conflict of interest.

## AUTHOR CONTRIBUTIONS

**Ziyuan Zhou:** Conceptualization (lead); formal analysis (lead); methodology (lead); visualization (lead); writing – original draft (lead). **Minghan Yu:** Data curation (lead); methodology (equal); project administration (equal); resources (supporting); supervision (lead); writing – original draft (supporting). **Guodong Ding:** Conceptualization (equal); funding acquisition (lead); methodology (equal); project administration (lead); resources (lead); supervision (lead); writing – original draft (equal). **Guanglei Gao:** Conceptualization (supporting); data curation (lead); methodology (supporting); project administration (equal); resources (equal); supervision (equal). **Yingying He:** Methodology (supporting); project administration (equal); resources (equal); writing – original draft (equal). **Genzhu Wang:** Methodology (supporting); writing – original draft (supporting).

## Supporting information

Figure S1

Table S1‐S3

## Data Availability

The data that support the findings of this study have been deposited in Dryad with https://doi.org/10.5061/dryad.m37pvmczk.
