# Supplementary figures and images for: Effects of Hedysarum leguminous plants on soil bacterial communities in the Mu Us Desert, northwest China
Source: Ecol Evol. 2020 Sep 21;10(20):11423–39. doi: 10.1002/ece3.6779 (PMC7593153; doi:10.1002/ece3.6779)

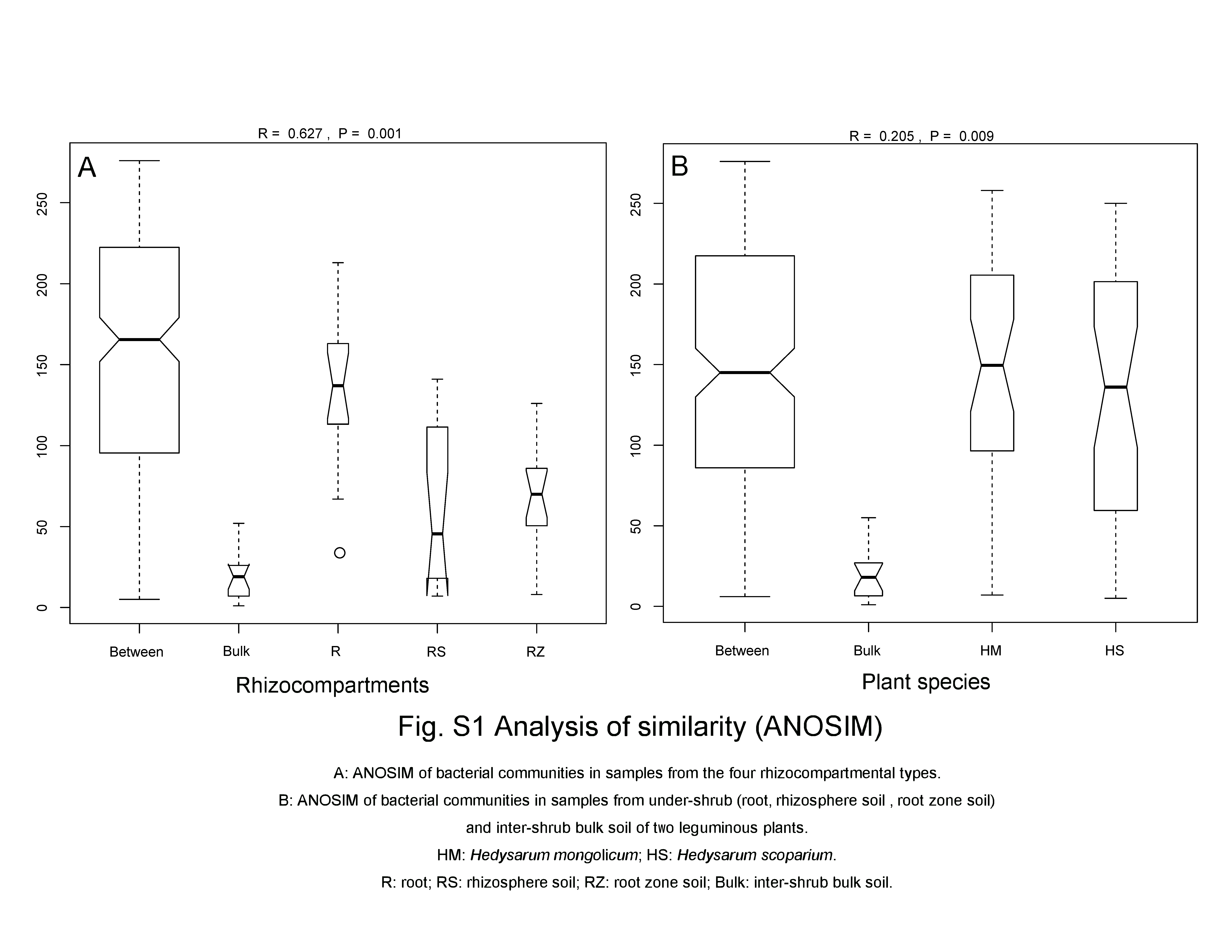

Supplement: Supplementary file 1 — Figure S1 [file ECE3-10-11423-s001.tiff]
