# Supplementary material for: Effects of Hedysarum leguminous plants on soil bacterial communities in the Mu Us Desert, northwest China
Source: Ecol Evol. 2020 Sep 21;10(20):11423–39. doi: 10.1002/ece3.6779 (PMC7593153; doi:10.1002/ece3.6779)
Supplement: Supplementary file 2 — Table S1‐S3 [file ECE3-10-11423-s002.docx]

| **TABLE S1. Quality metrics of 16S rRNA High-throughput sequencing.** (*n=6*) | | | | | | | |
| --- | --- | --- | --- | --- | --- | --- | --- |
| **A.** Total number of reads and tag length before and after quality checking and trimming | | | | | | | |
| Total # of raw reads before QC | | | | 1330969 | | | |
| Total # of clean reads after QC | | | | 1131924 | | | |
| **B.** Assigned reads | | Root | Rhizosphere soil | Root zone soil | | Bulk soil | |
| Assigned # of raw reads before QC | | 273007 | 312053 | 294188 | | 451721 | |
| Assigned # of clean reads after QC | | 212314 | 257666 | 251475 | | 410469 | |
| Good's Coverage（mean ± SD) | | 96.70±0.31%a | 95.57±0.31%b | 95.71±0.33%b | | 95.89±0.65%b | |
| **C.** Length distribution of clean reads(bp) | | |  | Sequences |  |  | Percent |
| 0-200 |  |  |  | 61 |  |  | <0.01% |
| 200-260 |  |  |  | 364 |  |  | 0.03% |
| 260-320 |  |  |  | 250 |  |  | 0.02% |
| 320-360 |  |  |  | 11825 |  |  | 1.05% |
| 360-380 |  |  |  | 996644 |  |  | 88.05% |
| 380-400 |  |  |  | 122149 |  |  | 10.79% |
| 400-420 |  |  |  | 352 |  |  | 0.03% |
| 420-440 |  |  |  | 67 |  |  | <0.01% |
| 440-460 |  |  |  | 30 |  |  | <0.01% |
| 460-480 |  |  |  | 47 |  |  | <0.01% |
| 480-500 |  |  |  | 115 |  |  | 0.01% |
| 500-520 |  |  |  | 18 |  |  | <0.01% |
| 520-540 |  |  |  | 2 |  |  | <0.01% |
| 540-560 |  |  |  | 0 |  |  | 0.00% |
| 560-600 |  |  |  | 0 |  |  | 0.00% |
| Total |  |  |  | 1131924 |  |  | 100.00% |
| A: Total number of reads calculated from the 24 samples of all rhizocompartments before and after quality control (QC);  B: Sequencing number ssigned to each rhizocompartment, and coverage rate and observation depth of the reads database of each sample by the sequencing characterized by Good’s Coverage index (this index reflects whether a sequencing result can represent the actual distribution of microbes in a sample);  C: Length distribution of high-quality sequences (clean reads), which characterizes the extraction concentration and purity of bacterial genomic DNA. (n=6, i.e., 2 shrub species × 3 replicates).  Bulk soil: inter-shrub bulk soil | | | | | | | |

**TABLE S2: Analysis of the significance of differences in the mean relative abundances (±SE) of major contributing bacterial taxa in the four rhizocompartments of the two leguminous plants at phylum-order-genus levels.** Normal distributions and homoscedasticity of variances of the data were checked with the Kolmogorov-Smirnov test and was analyzed using either Bartlett’s test. Significant differences in the variance of parameters were evaluated with ANOVA and post-hoc comparisons were conducted by LSD tests. **HM**-*Hedysarum mongolicum*; **HS**-*Hedysarum scoparium*. Plant rhizocompartment effects showed P value. Significance level: *P* < 0.05.

| **HM-Top 10 Phyla** | **Root** | | **Rhizosphere soil** | | **Root zone soil** | | **Bulk** | | **Rhizocompartments effect** | |
| --- | --- | --- | --- | --- | --- | --- | --- | --- | --- | --- |
|  | **%± SE** | ***P*** | **%± SE** | ***P*** | **%± SE** | ***P*** | **%± SE** | ***P*** | **F** | ***P*** |
| **Proteobacteria** | 62.02±2.82% | a | 32.25±0.98% | b | 31.76±4.24% | b | 15.65±0.76% | c | **54.47** | **<0.01** |
| **Actinobacteria** | 20.51±0.51% | c | 36.4±6.39% | b | 42.58±4.76% | b | 58.45±1.72% | a | **14.76** | **<0.01** |
| **Bacteroidetes** | 10.73±2.36% | a | 10.25±5.55% | a | 3.4±0.73% | b | 0.59±0.11% | c | **4.75** | **0.03** |
| **Chloroflexi** | 0.99±0.13% | b | 7.80±2.32% | a | 5.35±1.34% | a | 6.66±0.52% | a | **4.77** | **0.03** |
| **Gemmatimonadetes** | 0.79±0.24% | d | 3.04±0.19% | c | 5.29±0.63% | b | 7.92±0.46% | a | **53.37** | **<0.01** |
| **Acidobacteria** | 1.53±0.93% | a | 3.04±0.59% | a | 2.86±0% | a | 2.42±0.24% | a | 1.44 | 0.30 |
| **Verrucomicrobia** | 0.3±0.16% | b | 0.7±0.24% | b | 2.22±0.45% | a | 1.11±0.11% | b | **9.16** | **<0.01** |
| **Firmicutes** | 0.25±0.07% | b | 0.45±0.21% | b | 0.87±0.12% | a | 1.06±0.08% | a | **8.00** | **<0.01** |
| **Planctomycetes** | 0.22±0.07% | a | 1.33±0.49% | a | 1.64±0.47% | a | 1.66±0.18% | a | 3.72 | 0.06 |
| **Saccharibacteria** | 1.58±1.06% | a | 1.32±0.27% | a | 1.16±0.46% | a | 0.2±0.04% | a | 1.03 | 0.43 |
| **Total** | 98.92% |  | 96.58% |  | 97.13% |  | 95.72% |  |  |  |
| **HS-Top 10 Phyla** | **Root** | | **Rhizosphere soil** | | **Root zone soil** | | **Bulk** | | **Rhizocompartments effect** | |
|  | **%± SE** | ***P*** | **%± SE** | ***P*** | **%± SE** | ***P*** | **%± SE** | ***P*** | **F** | ***P*** |
| **Proteobacteria** | 53.81±2.54% | a | 34.95±3.24% | b | 25.87±1.93% | c | 22.2±0.50% | c | **38.17** | **<0.01** |
| **Actinobacteria** | 35.4±3.03% | b | 41.16±5.03% | a | 48.86±2.97% | a | 55.93±1.98% | a | **6.80** | **0.01** |
| **Bacteroidetes** | 4.75±0.75% | a | 3.29±0.61% | a | 3.85±2.56% | a | 1.64±0.34% | a | **0.90** | **0.48** |
| **Saccharibacteria** | 0.85±0.43% | a | 0.43±0.10% | a | 0.60±0.22% | a | 0.51±0.10% | a | **0.52** | **0.68** |
| **Acidobacteria** | 0.77±0.25% | a | 3.14±0.92% | a | 2.33±0.49% | a | 1.94±0.36% | a | **3.07** | **0.09** |
| **Chloroflexi** | 1.48±0.39% | c | 6.46±1.14% | a | 8.39±0.22% | a | 5.89±0.72% | b | **16.84** | **<0.01** |
| **Gemmatimonadetes** | 0.50±0.09% | c | 4.85±0.41% | a | 4.18±0.61% | b | 5.92±0.54% | a | **26.17** | **<0.01** |
| **Verrucomicrobia** | 0.35±0.15% | b | 0.97±0.17% | a | 0.69±0.10% | a | 0.84±0.02% | a | **4.67** | **0.04** |
| **Planctomycetes** | 0.59±0.04% | a | 0.64±0.03% | a | 0.76±0.23% | a | 0.80±0.33% | a | **0.25** | **0.86** |
| **Firmicutes** | 0.22±0.05% | b | 0.89±0.05% | b | 1.79±0.18% | a | 1.18±0.40% | a | **8.54** | **<0.01** |
| **Total** | 98.72% |  | 96.78% |  | 97.32% |  | 96.85% |  |  |  |
| **HM-Top 10 Orders** | **Root** | | **Rhizosphere soil** | | **Root zone soil** | | **Bulk** | | **Rhizocompartments effect** | |
|  | **%± SE** | ***P*** | **%± SE** | ***P*** | **%± SE** | ***P*** | **%± SE** | ***P*** | **F** | ***P*** |
| **Rhizobiales** | 21.97±1.64% | a | 10.76±0.96% | b | 9.27±1.80% | b | 3.1±0.14% | c | **35.89** | **<0.01** |
| **Burkholderiales** | 14.37±0.45% | a | 4.08±1.31% | b | 3.12±0.57% | b | 0.57±0.06% | c | **65.54** | **<0.01** |
| **Sphingomonadales** | 11.1±5.54% | a | 3.47±0.41% | a | 2.34±0.52% | a | 0.68±0.10% | b | **2.73** | **0.11** |
| **Caulobacterales** | 3.61±1.42% | a | 0.94±0.15% | b | 0.58±0.11% | b | 0.37±0.18% | b | 4.36 | 0.04 |
| **Propionibacteriales** | 2.49±0.09% | a | 3.17±0.22% | a | 2.71±0.24% | a | 1.59±0.09% | b | 14.61 | <0.01 |
| **Acidimicrobiales** | 1.03±0.14% | b | 3.84±0.57% | a | 4.57±0.38% | a | 4.96±0.31% | a | **21.45** | **<0.01** |
| **Nitrospirales** | 0.05±0.02% | b | 0.37±0.07% | b | 0.9±0.22% | a | 0.78±0.11% | a | **9.39** | **<0.01** |
| **Gaiellales** | 0.79±0.06% | b | 4.16±1.63% | b | 7.95±3.43% | b | 18.65±1.6% | a | **14.13** | **<0.01** |
| **Sphingobacteriales** | 4.38±0.67% | a | 7.75±4.58% | a | 1.73±0.67% | a | 0.26±0.10% | a | 1.98 | 0.20 |
| **Flavobacteriales** | 4.35±3.24% | a | 1.17±0.67% | a | 0.14±0.08% | a | - | a | 1.50 | 0.28 |
| **Total** | **64.14%** |  | **38.54%** |  | **33.31%** |  | **30.96%** |  |  |  |
| **HS-Top 10 Orders** | **Root** | | **Rhizosphere soil** | | **Root zone soil** | | **Bulk** | | **Rhizocompartments effect** | |
|  | **%± SE** | ***P*** | **%± SE** | ***P*** | **%± SE** | ***P*** | **%± SE** | ***P*** | **F** | ***P*** |
| **Rhizobiales** | 28.12±1.97% | a | 10.44±1.11% | b | 8.13±1.31% | b | 6.25±0.53% | b | **57.10** | **<0.01** |
| **Xanthomonadales** | 8.44±1.72% | a | 2.26±0.39% | b | 1.52±0.37% | b | 1.31±0.20% | b | 14.01 | <0.01 |
| **Micromonosporales** | 6.18±3.38% | a | 1.8±0.42% | a | 2.31±0.31% | a | 1.96±0.33% | a | 1.48 | 0.29 |
| **Micrococcales** | 5.19±1.37% | a | 4.56±0.45% | a | 5.07±1.17% | a | 3.14±0.46% | a | 0.97 | 0.45 |
| **Caulobacterales** | 5.03±0.88% | a | 0.67±0.20% | b | 0.40±0.10% | b | 0.57±0.12% | b | **24.12** | **<0.01** |
| **Burkholderiales** | 3.42±0.83% | a | 4.78±1.63% | a | 2.02±0.23% | a | 1.25±0.21% | b | 2.82 | 0.10 |
| **Propionibacteriales** | 2.18±0.53% | a | 3.99±0.43% | a | 3.08±0.6% | a | 2.48±0.11% | a | 3.03 | 0.09 |
| **Sphingomonadales** | 1.87±0.83% | a | 1.75±0.33% | a | 1.85±1.33% | a | 0.81±0.4% | a | **0.38** | **0.77** |
| **Gaiellales** | 1.01±0.14% | b | 6.77±2.23% | b | 11.22±3.08% | a | 15.46±0.31% | a | **10.52** | **<0.01** |
| **Nitrosomonadales** | 0.28±0.06% | a | 4.07±0.96% | a | 3.26±0.81% | a | 3.27±0.16% | a | 6.99 | 0.01 |
| **Total** | 61.72% |  | 41.09% |  | 38.86% |  | 36.50% |  |  |  |
| **HM-Top 10 Genera** | **Root** | | **Rhizosphere soil** | | **Root zone soil** | | **Bulk** | | **Rhizocompartments effect** | |
|  | **%± SE** | ***P*** | **%± SE** | ***P*** | **%± SE** | ***P*** | **%± SE** | ***P*** | **F** | ***P*** |
| ***Sphingomonas*** | 9.22±6.04% | a | 1.98±0.23% | a | 1.29±0.38% | a | 0.33±0.03% | a | **1.80** | **0.22** |
| ***Rhizobium*** | 8.31±0.70% | a | 1.43±0.75% | b | 0.55±0.20% | b | 0.07±0.02% | b | **53.98** | **<0.01** |
| ***Massilia*** | 3.94±0.15% | a | 0.32±0.14% | b | 0.09±0.01% | b | 0.02±0.01% | b | **325.43** | **<0.01** |
| ***Cupriavidus*** | 3.71±2.93% | a | 0.06±0.01% | a | 0.05±0.03% | a | 0.01±0.01% | a | **1.57** | **0.27** |
| ***Bosea*** | 2.21±1.40% | a | 0.3±0.07% | a | 0.16±0.05% | a | 0.04±0.01% | a | **2.15** | **0.17** |
| ***Caulobacter*** | 2.33±1.22% | a | 0.16±0.02% | a | 0.05±0.02% | a | 0.01±0.01% | a | **3.45** | **0.07** |
| ***Flavobacterium*** | 1.79±1.01% | a | 0.87±0.66% | a | 0.07±0.03% | a | - | a | **1.93** | **0.20** |
| ***Mycobacterium*** | 1.65±0.14% | a | 1.49±0.23% | a | 0.87±0.19% | b | 0.6±0.08% | b | **8.67** | **<0.01** |
| ***Gaiella*** | 0.21±0.03% | b | 1.65±0.62% | b | 2.51±0.68% | b | 5.29±0.98% | a | **10.17** | **<0.01** |
| ***Roseiflexus*** | 0.16±0.04% | a | 2.21±0.88% | a | 1.92±1.01% | a | 1.4±0.29% | a | **1.76** | **0.23** |
| **Total** | 33.53% |  | 10.47% |  | 7.56% |  | 7.77% |  |  |  |
| **HS-Top 10 Genera** | **Root** | | **Rhizosphere soil** | | **Root zone soil** | | **Bulk** | | **Rhizocompartments effect** | |
|  | **%± SE** | ***P*** | **%± SE** | ***P*** | **%± SE** | ***P*** | **%± SE** | ***P*** | **F** | ***P*** |
| ***Rhizobium*** | 5.72±2.32% | a | 0.93±0.38% | b | 0.35±0.25% | b | 0.38±0.17% | b | **4.80** | **0.03** |
| ***Steroidobacter*** | 4.82±1.29% | a | 0.33±0.06% | b | 0.17±0.03% | b | 0.36±0.09% | b | **12.25** | **0.02** |
| ***Phyllobacterium*** | 4.29±1.09% | a | 0.37±0.06% | b | 0.52±0.39% | b | 0.1±0.03% | b | **11.74** | **0.03** |
| ***Mycobacterium*** | 4.06±1.5% | a | 1.06±0.3% | a | 1.16±0.56% | a | 0.93±0.05% | a | **3.43** | **0.07** |
| ***Caulobacter*** | 3.95±0.79% | a | 0.17±0.05% | b | 0.1±0.08% | b | 0.14±0.07% | b | **22.97** | **<0.01** |
| ***Streptomyces*** | 4.06±1.46% | a | 1.28±0.13% | a | 1.31±0.29% | a | 0.99±0.14% | a | **3.69** | **0.06** |
| ***Bradyrhizobium*** | 2.64±0.82% | a | 0.5±0.06% | a | 0.28±0.06% | b | 0.31±0.05% | b | **7.67** | **0.01** |
| ***Promicromonospora*** | 2.51±1.58% | a | 0.27±0.15% | a | 0.08±0.02% | a | 0.22±0.11% | a | **2.14** | **0.17** |
| ***Variibacter*** | 2.36±0.24% | a | 0.89±0.12% | b | 0.62±0.1% | b | 0.49±0.05% | b | **36.15** | **<0.01** |
| ***Gaiella*** | 0.32±0.04% | b | 2.79±0.94% | a | 4.03±1.12% | a | 4.54±0.54% | a | **5.80** | **0.02** |
| **Total** | 32.37% |  | 8.59% |  | 8.62% |  | 8.46% |  |  |  |

**TABLE S3:** **The significance test data of the CCA/RDA model, axes, and terms.**

**FIGURE 7A**: The significance test data of the CCA/RDA model, axes, and terms.

Analysis 'Interactive-forward-selection', step 'Forward Selection'

Method: CCA

Total variation is 2.83271, explanatory variables account for 39.2%

(adjusted explained variation is 6.7%)

| **FIGURE 7A: Summary Table.** | | | | |
| --- | --- | --- | --- | --- |
| **Statistic** | **Axis 1** | **Axis 2** | **Axis 3** | **Axis 4** |
| **Eigenvalues** | 0.358 | 0.1728 | 0.1288 | 0.1034 |
| **Explained variation (cumulative)** | 14.01 | 20.78 | 25.32 | 28.97 |
| **Pseudo-canonical correlation** | 0.9183 | 0.8581 | 0.8108 | 0.9057 |
| **Explained fitted variation (cumulative)** | 35.79 | 53.08 | 64.69 | 74.02 |

| **FIGURE 7A: Forward Selection Results.** | | | | |
| --- | --- | --- | --- | --- |
| **Name** | **Explains (%)** | **Contribution (%)** | **pseudo-F** | **P** |
| **TN** | 12.8 | 32.6 | 3.2 | 0.002 |
| **SWC** | 6 | 15.4 | 1.6 | 0.012 |
| **NH_4_^+^** | 4.6 | 11.7 | 1.2 | unknown |
| **NO_3_^-^** | 3.7 | 9.4 | 1 | 0.514 |
| **TP** | 3.8 | 9.6 | 1 | 0.46 |
| **SOC** | 3.3 | 8.3 | 0.8 | 0.69 |
| **AP** | 2.3 | 5.9 | 0.6 | 0.918 |
| **pH** | 2.7 | 6.9 | 0.7 | 0.838 |

**FIGURE 7B**: The significance test data of the CCA/RDA model, axes, and terms.

Analysis 'Interactive-forward-selection', step 'Forward Selection'

Method: RDA

Total variation is 0.10257, explanatory variables account for 42.1%

(adjusted explained variation is 11.2%)

| **FIGURE 7B: Summary Table.** | | | | | | | | |
| --- | --- | --- | --- | --- | --- | --- | --- | --- |
| **Statistic** | | | **Axis 1** | | **Axis 2** | **Axis 3** | | **Axis 4** |
| **Eigenvalues** | | | 0.2937 | | 0.0922 | 0.0181 | | 0.0071 |
| **Explained variation (cumulative)** | | | 29.37 | | 38.59 | 40.4 | | 41.11 |
| **Pseudo-canonical correlation** | | | 0.7125 | | 0.6262 | 0.6517 | | 0.4676 |
| **Explained fitted variation (cumulative)** | | | 69.74 | | 91.63 | 95.93 | | 97.62 |
| **FIGURE 7B: Forward Selection Results.** | | | | | | | | |
| **Name** | **Explains (%)** | **Contribution (%)** | | **pseudo-F** | | | **P** | |
| **TN** | 25.3 | 60.1 | | 7.5 | | | 0.002 | |
| **SWC** | 6.9 | 16.4 | | 2.1 | | | 0.084 | |
| **NH_4_^+^** | 2.8 | 6.7 | | 0.9 | | | 0.442 | |
| **NO_3_^-^** | 2.3 | 5.5 | | 0.7 | | | 0.538 | |
| **TP** | 2.1 | 4.9 | | 0.6 | | | 0.62 | |
| **SOC** | 1.1 | 2.5 | | 0.3 | | | 0.924 | |
| **AP** | 1.1 | 2.7 | | 0.3 | | | unknown | |
| **pH** | 0.5 | 1.1 | | 0.1 | | | 0.982 | |
